# Performance evaluation of tuberculosis smear microscopists working at rechecking laboratories in Ethiopia

**DOI:** 10.4102/ajlm.v6i1.590

**Published:** 2017-04-21

**Authors:** Habtamu Asrat, Abebaw Kebede, Abnet Abebe, Abyot Meaza, Getinet Hailu, Adinew Desale, Andargachew Gashu, Wondwossen Kassa, Tesfaye Mekonnen, Ebisea Abose, Feven Girmachew, Dereje Yenealem, Achamyeleh Mulugeta, Gonfa Ayana, Kassu Desta

**Affiliations:** 1 Ethiopian Public Health Institute, Addis Ababa, Ethiopia; 2 Department of Medical Laboratory Sciences, College of Health Sciences, Addis Ababa University, Addis Ababa, Ethiopia

## Abstract

**Background:**

Tuberculosis is an infectious disease caused by the bacillus *Mycobacterium tuberculosis*. According to the Ethiopian Federal Ministry of Health’s 2013–2014 report, the tuberculosis case detection rate was 53.7%, which was below the target of 81% set for that year.

**Objective:**

This study assessed the performance of tuberculosis smear microscopists at external quality assessment rechecking laboratories in Ethiopia.

**Methods:**

A cross-sectional study was conducted at 81 laboratories from April to July 2015. Panel slides were prepared and validated at the National Tuberculosis Reference Laboratory. The validated panel slides were used to evaluate the performance of microscopists at these laboratories compared with readers from the reference laboratory.

**Results:**

A total of 389 external quality assessment rechecking laboratory microscopists participated in the study, of which 268 (68.9%) worked at hospitals, 241 (62%) had more than five years of work experience, 201 (51.7%) held Bachelors degrees, and 319 (82%) reported tuberculosis smear microscopy training. Overall, 324 (83.3%) participants scored ≥ 80%. Sensitivity for detecting tuberculosis bacilli was 84.5% and specificity was 93.1%. The overall percent agreement between participants and reference readers was 87.1 (kappa=0.72). All 10 slides were correctly read (i.e., scored 100%) by 80 (20.6%) participants, 156 (40.1%) scored 90% – 95%, 88 (22.6%) scored 80% – 85% and 65 (16.7%) scored below 80%. There were 806 (20.7%) total errors, with 143 (3.7%) major and 663 (17%) minor errors.

**Conclusion:**

The overall performance of participants in reading the slides showed good agreement with the reference readers. Most errors were minor, and the ability to detect tuberculosis bacilli can be improved through building the capacity of professionals.

## Introduction

Tuberculosis is an infectious disease caused by the bacillus *Mycobacterium tuberculosis*. It remains a major global health problem, responsible for ill health among millions of people each year.^1,2,3^ It is the second leading cause of death among all infectious diseases worldwide after HIV.^1,3^ According to the 2014 World Health Organization Global Tuberculosis Report, there were nine million new tuberculosis cases and 1.5 million tuberculosis deaths (1.1 million among HIV-negative people and 0.4 million among HIV-positive people) in 2013.^1^ One quarter of global cases and deaths occurred in the African Region,^1^ and Ethiopia ranked 10th in tuberculosis incidence among 22 high-burden countries.^1,4^

According to Ethiopian Ministry of Health reports for 2012–2013 and 2013–2014, the targeted tuberculosis case detection rates were 82.7% for the 2012–2013 period and 81% for 2013–2014. However, the case detection rates achieved were 58.9% in 2012–2013 and 53.7% in 2013–2014, which were well below these targets.^5,6,7^ A low case detection rate is often associated with a lack of effective programme awareness, lack of active cough identification and lack of quality-assured routine diagnosis (such as sputum quality, reagent quality, knowledge, and capacity of professionals). In Ethiopia, factors that are associated with low case detection rates have not been well studied. However, they are likely to be associated with these factors. Therefore, the present study dealt with the performance of tuberculosis smear microscopists as one factor affecting quality-assured routine diagnosis.

In most low- and middle-income countries, smear microscopy remains the foundation of tuberculosis diagnosis, despite its relatively low sensitivity. Microscopy has also remained essential to monitoring of tuberculosis treatment. A microscopy network with adequate population coverage and high quality performance is therefore critical. Bright-field sputum smear microscopy (i.e., conventional Ziehl-Neelsen staining) is widely available, simple to perform, inexpensive, and requires simple laboratory facilities.^1,8^ Thus, one national tuberculosis control strategy recommended by the World Health Organization is to pursue expansion and enhancement of high-quality directly-observed treatment, short-course chemotherapy through early case detection and diagnosis at quality-assured laboratories.^9^

Quality assurance of microscopy remains a critical activity of all laboratory networks, and a comprehensive external quality assessment (EQA) programme that includes on-site evaluation, random blinded rechecking, and panel testing should be implemented.^1,8,10^ EQA programmes are needed to ensure that smears are performed and stained properly, results are interpreted correctly and all microscopy centres achieve an acceptable level of performance. Effective EQA programmes require dedicated and qualified staff for rechecking of smears. The implementation of EQA for microscopy has the advantage, not only of strengthening laboratory networks, but of improving diagnostic quality.^11^

The Ethiopian Federal Ministry of Health and the Ethiopian Public Health Institute decentralised EQA programmes to regional reference laboratories and have guided the regions to decentralise further into sub-regional laboratories and EQA rechecking laboratories. This decision was made with the assumption that all microscopy centres in the various regions would have a chance to participate in EQA programmes and that improved EQA coverage could be achieved. The mandate for conducting a rechecking programme was given to the EQA rechecking laboratories by Regional Health Bureaus. Following the endorsement of the Regional Health Bureaus, EQA rechecking laboratories have the right to perform tuberculosis EQA blind rechecking by collecting slides from the microscopy centres in their catchment areas. The aim of this study was to produce baseline data about the performance of the tuberculosis rechecking laboratories and the microscopists who work there.

## Methods

### Ethical considerations

Leftover samples were collected anonymously from federal hospitals for panel preparation. All information about each tuberculosis EQA rechecking laboratory was kept confidential and used only for the purposes of this study and for the improvement of acid-fast bacilli (AFB) microscopy. The research proposal was evaluated and approved by the Departmental Research and Ethics Review Committee of the Department of Medical Laboratory Sciences, College of Health Sciences at Addis Ababa University with Ref. No. MLS/326/15 and Protocol number: DRERC 119/15/MLS before the start of fieldwork. Confidentiality was maintained during data collection, and written informed consent was obtained from each study participant.

### Study design

A cross-sectional study was conducted from April to July 2015 at tuberculosis EQA rechecking laboratories in Ethiopia. Validated panel slides were used to assess the performance of microscopists working in the laboratories. The study was conducted at 12 (100%) regional laboratories, three (75%) sub-regional laboratories, 46 (61%) hospital laboratories and 20 (59%) health centre laboratories among the 125 EQA rechecking laboratories in Ethiopia.

Sputum samples for panel preparation were collected from federal hospitals anonymously, and panel slides were prepared in the National Tuberculosis Reference Laboratory. Each dilution panel was validated by six different readers following World Health Organization guidelines (reference readers).^10^ A set of 10 validated slides was distributed to participating laboratories to assess the reading and interpretation proficiency of smear microscopists; 50–70 minutes were allowed to complete the reading.^10,12^ The panel composition and bacilli load were: one grade 3+ slide, one grade 2+ slide, two grade 1+ slides, three 1–9 AFB/100 field slides and three negative slides.^10^

The results were expressed as correct, minor error or major error. Major errors were classified as high false positive, if a negative smear was misread as grade 1+ to 3+ positive, or as high false negative, if a grade 1+ to 3+ positive smear was misread as negative (Table 1). Minor errors were classified as a quantification error, when there was a difference of more than one grade in the reading of positive smear between the examinee and the reference readers, a low false positive, when a negative smear was misread as scanty (1–9 AFB/100x field), or as low false negative, when a scanty slide (1–9 AFB/100x field) was misread as negative.^10,12,13^

**TABLE 1:** Evaluation and interpretation of errors between rechecking laboratory microscopists and reference readers, Ethiopia, April–July 2015†.

Result of microscopist	Result of reference readers				
	Negative	1-9 AFB / 100 fields	1+	2+	3+
Negative	Correct	LFN	HFN	HFN	HFN
1-9 AFB/100 fields	LFP	Correct	Correct	QE	QE
1+	HFP	Correct	Correct	Correct	QE
2+	HFP	QE	Correct	Correct	Correct
3+	HFP	QE	QE	Correct	Correct

HFN, high false negative; HFP, high false positive; LFN, low false negative; LFP, low false positive; QE, quantification error.

† Reference readers were microscopists at Ethiopia’s National Tuberculosis Reference Laboratory.

Each slide was worth 10 points. The total possible score was 100 points (for 10 slides), and based on national and World Health Organization guidelines, a passing score was considered to be ≥ 80%.^10,12^ Committing major errors, like a high false positive or high false negative, was worth zero points, whereas minor errors, like low false positive, low false negative and quantification errors, were worth five points.^8,10,12^

### Data analysis

All data were entered into a Microsoft Excel (Microsoft, Inc., Redmond, Washington, United States) spreadsheet and transported to SPSS (version 20.0; SPSS, Inc., Chicago, Illinois, United States) for analysis. The percentages of agreements, differences and the different types of errors were calculated. The sensitivity, specificity, positive predictive value, and negative predictive value of smear reading for each tuberculosis EQA smear microscopist was calculated. The Chi square test was used to assess associations between different variables. The strength of an agreement between participant readers and the reference readers were assessed using kappa statistics.^14^

## Results

### Study participants

A total of 389 microscopists (2 to 13 microscopists per rechecking laboratory) from 81 tuberculosis EQA rechecking laboratories participated in the study, of whom 263 (67.6%) were men and 126 (32.4%) were women (Table 2). Most of the study participants worked in hospital laboratories (*n* = 268, 68.9%); 241 (62%) participants had more than five years of work experience in tuberculosis smear microscopy services, 201 (51.7%) held a Bachelors degree, and 319 (82%) had gone through tuberculosis smear microscopy in-service training.

**TABLE 2:** Demographic characteristics of microscopists at tuberculosis external quality assessment rechecking laboratories in Ethiopia (*N* = 389), April–July, 2015.

Variables	Frequency	
	Number	Percent
**Sex**		
Male	263	67.6
Female	126	32.4
**Place of work**		
Regional or sub-regional laboratory	62	15.9
Hospital	268	68.9
Health centre	59	15.2
**Work experience**		
< 2 years	23	5.9
2–5 years	125	32.1
> 5 years	241	62.0
**Educational background**		
Diploma	169	43.4
Bachelors degree	201	51.7
Masters degree	19	4.9
**Tuberculosis smear microscopy in-service training**		
Yes	319	82.0
No	70	18.0

### Panel testing

Among all 389 participants, 324 (83.3%) scored ≥ 80% (passing) (Table 3). When stratified by place of work, more participants working in hospitals (*n* = 231, 86.2%) achieved a passing score (≥ 80 %) than participants who worked in other types of facilities. On the other hand, 21/23 (91.3%) microscopists with less than two years of work experience scored ≥ 80%, which was higher than microscopists with more than two years of work experience. The proportion of participants who scored ≥ 80% was higher among holders of a Masters degree compared with participants with other educational backgrounds, and the proportion of participants who scored ≥ 80% was slightly higher among participants who had not had tuberculosis smear microscopy in-service training. In general, there were no statistically-significant associations between the performance of participants in tuberculosis bacilli detection and their sex, work experience, educational background, place of work or tuberculosis smear microscopy in-service training.

**TABLE 3:** Relationship between demographic characteristics and scores of microscopists at tuberculosis external quality assessment rechecking laboratories in Ethiopia (*N* = 389), April–July, 2015.

Variable	Passed ≥ 80% No. (%)	Failed < 80% No. (%)	Chi-square	Degree of freedom	*P*-value
**Sex**					
Male	221 (84.0)	42 (16.0)	0.319	1	0.572
Female	103 (81.7)	23 (18.3)			
**Place of work**					
Regional or sub-regional laboratory	49 (79)	13 (21)			
Hospital	231 (86.2)	37 (13.8)	5.650	2	0.059
Health centre	44 (74.6)	15 (25.4)			
**Work experience**					
< 2 years	21 (91.3)	2 (8.7)			
2–5 years	107 (85.6)	18 (14.4)	2.207	2	0.332
> 5 years	196 (81.3)	45 (18.7)			
**Educational background**					
Diploma	136 (80.5)	33 (19.5)			
Bachelors degree	171 (85.1)	30 (14.9)	1.945	2	0.378
Masters degree	17 (89.5)	2 (10.5)			
**Tuberculosis smear microscopy in-service training**					
Yes	265 (83.1)	54 (16.9)	0.061	1	0.805
No	59 (84.3)	11 (15.7)			
Overall performance	324 (83.3)	65 (16.7)			

A total of 3890 validated slides were read by study participants (Table 4). The overall sensitivity for detecting tuberculosis bacilli was 84.5% and overall specificity was 93.1%. The overall percent agreement of participants with the reference readers was 87.1 (kappa=0.72). The percent agreement of participants working in health centres with the reference readers was 83.1% (kappa=0.64), which was slightly lower than participants working in hospitals or regional laboratories. The negative predictive values were quite low for participants working in all health facilities.

**TABLE 4:** Overall sensitivity, specificity, predictive values and agreements of participants with reference readers in detecting tuberculosis bacilli, Ethiopia, April–July, 2015.

EQA rechecking laboratory microscopists		Reference readers		Total	Sensitivity (%)	Specificity (%)	PPV	NPV	Percent agreement	Kappa
		Positive	Negative							
Type of facility										
Regional or sub-regional laboratory	Positive	376	16	392	86.6	91.4	95.9	74.6	88.1	0.73
	Negative	58	170	228						
	Total	434	186	620						
Hospital	Positive	1598	51	1649	85.2	93.7	96.9	73.0	87.7	0.73
	Negative	278	753	1031						
	Total	1876	804	2680						
Health centre	Positive	326	13	339	78.9	92.7	96.2	65.3	83.1	0.64
	Negative	87	164	251						
	Total	413	177	590						
Overall	Positive	2300	80	2380	84.5	93.1	96.6	72.0	87.1	0.72
	Negative	423	1087	1510						
	Total	2723	1167	3890						

EQA, external quality assessment; NPV, negative predictive value; PPV, positive predictive value.

**FIGURE 1:**
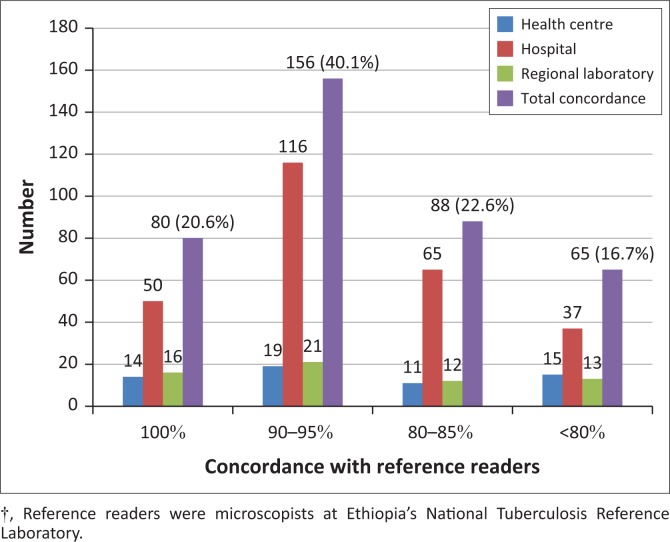
Concordance of tuberculosis external quality assessment rechecking laboratory microscopists with reference readers for detecting tuberculosis bacilli, Ethiopia (*N*=389), April–July, 2015†.

Of the 389 participants, 80 (20.6%) participants correctly read all 10 slides and scored 100% (Figure 1). A total of 156 (40.1%) scored 90% – 95%, which means they committed one major error or two minor errors. A total of 88 (22.6%) participants scored 80% – 85%, which means they committed three to four minor errors or two major errors or one major and one minor error or one major and two minor errors. Finally, 65 (16.7%) participants scored below 80%, which means they had more than four minor errors or two major errors or one major and two minor errors.

Of the 3890 examined slides, there were a total of 806 (20.7%) errors, which included 143 (3.7%) major errors and 663 (17%) minor errors (Table 5). Of these, 89 (2.3%) errors were high false negatives, 54 (1.4%) were high false positives, 334 (8.6%) were low false negatives, 26 (0.7%) were low false positives and 303 (7.8%) were quantification errors.

**TABLE 5:** Type of errors committed by external quality assessment rechecking laboratory microscopists in detecting tuberculosis bacilli by type of institution, Ethiopia (*N*=3890 slides), April–July, 2015.

Type of health facility	Major error		Minor error			Total errors
	HFN No. (%)	HFP No. (%)	LFN No. (%)	LFP No. (%)	QE No. (%)	No. (%)
Regional or sub-regional laboratory (*n* = 620)	11 (1.8)	11 (1.8)	47 (7.6)	5 (0.8)	75 (12.1)	149 (24)
Hospital (*n* = 2680)	62 (2.3)	31 (1.2)	216 (8.1)	20 (0.7)	194 (7.2)	523 (19.5)
Health centre (*n* = 590)	16 (2.7)	12 (2.0)	71 (12)	1 (0.2)	34 (5.8)	134 (22.7)
Total	89 (2.3)	54 (1.4)	334 (8.6)	26 (0.7)	303 (7.8)	806 (20.7)
	143 (3.7)			663 (17)			

HFN, high false negative; HFP, high false positive; LFN, low false negative; LFP, low false positive; QE, quantification error.

## Discussion

This cross-sectional study evaluated the performance of tuberculosis smear microscopists working at EQA rechecking laboratories and the status of the respective laboratories. In this study, the overall agreement of participants with reference readers for reading the validated slides was 87.1% (kappa=0.72), which was good agreement based on kappa statistics.^14^ However, lower agreement was observed when compared with a different study conducted in Southern Ethiopia, which found 96.8% agreement (kappa=0.936),^15^ and a study done in the town of Hawassa, Ethiopia, which found 95.2% agreement (kappa=0.73).^16^ When compared with a study done in the East and West Amhara regions of Ethiopia, higher agreement was also observed (98.4% in East Amhara and 96.5% in West Amhara [kappa=0.92]).^17,18^ Thus, performance in our study was slightly lower than in similar studies conducted in other parts of Ethiopia. This may have been due to the large number of laboratories and/or laboratory professionals included in our study, which was more of a nationwide study with wider representation. This may have made our study more prone to lower performance.

In general, agreement in reading between participants and reference readers was slightly lower than in similar studies conducted in other countries. Our finding was lower than studies done in India (98% agreement)^19^ and Tanzania^20^ (89.2% agreement). However, agreement in our study was higher than studies done in Ghana (73%)^21^ and the Democratic Republic of Congo (74%).^22^ These differences may be attributable to differences in the composition of the panel slides, as we prepared a second degree of difficulty in our slides (three scanty and three negative slides).^10^

In our study, the overall sensitivity was 84.5% and specificity was 93.1%. The study in Hawassa, Ethiopia showed higher sensitivity (91.97%), but lower specificity (80.0%).^16^ On the other hand, both sensitivity (96.5%) and specificity (96.4%) were higher in the West Amhara, Ethiopia report.^18^ Similarly, the study conducted in East Amhara, Ethiopia showed higher sensitivity (88.4%) and specificity (99.3%).^17^ Both sensitivity and specificity were 96.8% in a Southern Ethiopia finding, which was higher than our study.^15^ Sensitivity (88.5%) and specificity (100%) were also higher in the study conducted in Tanzania.^20^ In our study, the lower sensitivity indicates that there were high false-negative rates (patients with tuberculosis bacilli misdiagnosed as negative). The consequence of this low sensitivity is that tuberculosis patients are not treated, which results in ongoing disease, disease transmission or death.

The study in Hawassa, Ethiopia reported that 13.6% of microscopists correctly read all panel slides, which was slightly lower than our finding, and 86.4% committed at least one error in reading 10 slides, which was significantly higher than our finding.^16^ In the study done in India, 95% of readers reported no errors,^19^ demonstrating far greater proficiency than the present study. Although the majority of our participants (83.3%) had an acceptable performance score (≥ 80%), we consider this a weak achievement, since study participants were from facilities with a responsibility to recheck other health institutions’ slides and provide support to them. Considering this responsibility, microscopists at these facilities should have scored better than the current findings.

In the present study, there were more low false negatives than quantification errors. False readings in Southern Ethiopia (3.2%), East Amhara (1.6%) and West Amhara (3.5%), Ethiopia were lower than our finding.^15,17,18^ On the other hand, the percentage of errors in Hawassa, Ethiopia (29.75%) was higher than ours.^16^ In addition, the Hawassa study had fewer major errors (2.22%), but more minor errors (27.5%) than our study.^16^ Quantification errors were the biggest contributor to overall errors in the Hawassa study, whereas in the present study, low false negatives were the most frequent errors.^16^ Fewer errors were observed in India, where quantification errors were the most frequent and no high false positives were reported.^19^ In a similar study conducted in Mexico, quantification errors were frequent (12.3%), followed by low false negatives (5.7%).^23^ A study conducted in the Democratic Republic of Congo also reported frequent low false-negative errors.^22^ In another study conducted in Taiwan, low false-positive errors were much higher (28.6%) than in the present study.^24^

False-negative errors could lead to failure to detect persons with infectious tuberculosis, who could continue to spread the disease in their communities; false positives could lead to unnecessary anxiety, exposure of patients to unwanted side effects of medications, and unnecessary expenditure.^25^ While lower rates of minor errors are acceptable due to the inherent problems of AFB smear microscopy services, major errors are unacceptable. Among minor errors, low false-positive and low false-negative errors both have a significant impact on patient management and tuberculosis control programmes, whereas quantification errors have no impact on patient management. Hence, improving the competency of professionals through training, implementation of strong EQA programmes, supportive supervision and mentoring are critically important to reduce or avoid these types of errors and to maximise case detection rates.^10^

### Limitations

This study has a few limitations, which should be considered when interpreting our results. First, unstained slides were not sent to participating laboratories. Thus, the quality of the reagents used for routine AFB microscopy was not assessed. Additionally, information on the age of the participants was not collected in the demographic information. Therefore, we could not evaluate the effects of age variability on the performance of the study participants.

### Conclusion

The overall performance of the tuberculosis EQA rechecking laboratories in reading AFB slides showed good agreement with the reference readers (87.1%). Overall, 20.7% of slides were misread, and most errors were minor. Nevertheless, these errors are alarming, and our findings are a clarion call to tuberculosis control programmes to give needed support to EQA programmes. A large number of minor errors were noted; thus, continuous mentoring and supportive supervision for AFB microscopy centres should be given priority to minimise errors and improve EQA activities in Ethiopia. Tuberculosis is a re-emerging global threat, and all steps to improve the accuracy of its diagnosis should be sought and implemented.
